# Hydropneumothorax as a Diagnostic Clue to Pulmonary Tuberculosis With Pleural Involvement: A Case Report

**DOI:** 10.7759/cureus.117754

**Published:** 2026-10-11

**Authors:** Sara Bravo, Sérgio Costa Monteiro, Raquel Vieira, Juliana Nogueira, Filipa Coroado Ferreira

**Affiliations:** 1 Internal Medicine, Unidade Local de Saúde da Região de Aveiro, Aveiro, PRT

**Keywords:** hydropneumothorax, pleural effusion, pleural tuberculosis, pulmonary tuberculosis, tuberculosis

## Abstract

Hydropneumothorax represents an unusual presentation of pulmonary tuberculosis (TB), posing diagnostic and therapeutic challenges. We report the case of a 36-year-old woman who presented with productive cough, dyspnea, right-sided pleuritic chest pain, and constitutional symptoms. Chest imaging revealed a large right-sided hydropneumothorax with extensive parenchymal and cavitary disease. Sputum smear microscopy was positive for acid-fast bacilli, and molecular testing of both sputum and pleural fluid detected *Mycobacterium tuberculosis* complex, confirming pulmonary TB with pleural involvement. Anti-TB therapy and chest tube drainage resulted in rapid and satisfactory re-expansion of the right lung. A small residual pneumothorax persisted after chest tube removal, without recurrence requiring further pleural drainage. This case highlights hydropneumothorax as an uncommon but clinically relevant manifestation of pulmonary TB and emphasizes the importance of considering TB in the differential diagnosis of spontaneous hydropneumothorax. Early recognition and appropriate management may contribute to favorable clinical outcomes.

## Introduction

Tuberculosis (TB) continues to represent a major global health concern, particularly in high-burden settings, with an estimated 10.7 million people developing the disease worldwide in 2024 [1]. Beyond its substantial epidemiological burden, TB remains clinically challenging because of its heterogeneous presentation, particularly when it manifests with uncommon radiological findings.

Pulmonary TB encompasses a broad spectrum of radiological manifestations, ranging from parenchymal consolidation, centrilobular and tree-in-bud nodules, and lymphadenopathy to miliary dissemination, pleural involvement, and cavitary disease [2]. Although cavitation is a well-recognized manifestation of post-primary pulmonary TB, its absence does not exclude active disease, which may present with a variety of non-cavitary or atypical imaging patterns [2].

Hydropneumothorax is an uncommon but well-described and potentially serious complication of pulmonary TB. It may arise from communication between a tuberculous pulmonary or pleural lesion and the pleural space, particularly in the setting of a bronchopleural fistula (BPF). Its occurrence may complicate the clinical course, frequently requiring prolonged pleural drainage or, in selected cases, surgical management [3]. Unlike a simple tuberculous pleural effusion, which consists of pleural fluid without an associated pleural air component, hydropneumothorax is characterized by the simultaneous presence of air and fluid within the pleural space, typically producing a horizontal air-fluid level. When the pleural fluid is infected or purulent, the condition may be termed pyopneumothorax. Chronic tuberculous pleural inflammation may also lead to visceral pleural fibrosis and a non-expandable or ‘trapped’ lung, potentially impairing lung re-expansion after pleural drainage. Tuberculous hydropneumothorax should be distinguished from other infectious and non-infectious causes of pleural air and fluid, including bacterial empyema, necrotizing pneumonia, malignancy, trauma, and iatrogenic causes. We report the case of a 36-year-old woman in whom hydropneumothorax was the initial presentation leading to the diagnosis of pulmonary TB with pleural involvement.

This work was previously presented in the “Images in Medicine” category at the 31st National Congress of Internal Medicine, held in Coimbra, Portugal, on May 23, 2025.

## Case presentation

We report the case of a 36-year-old woman, an active smoker, who presented to the emergency department with a one-week history of productive cough, dyspnea, right-sided pleuritic chest pain, and generalized malaise. She also reported anorexia, asthenia, and unquantified weight loss over the preceding month. She denied trauma, previous thoracic surgery, or *Mycobacterium tuberculosis* infection. On admission, she was tachycardic (heart rate, 117 beats/min), tachypneic (respiratory rate, 26 breaths/min), febrile (tympanic temperature, 39°C), and hypoxemic, with a peripheral oxygen saturation of 90% on room air. Her blood pressure was 126/78 mmHg. Pulmonary auscultation revealed absent breath sounds over the right hemithorax and scattered rhonchi on the left. Percussion of the right hemithorax demonstrated hyperresonance over the upper zone and dullness over the lower zone. Cardiac auscultation revealed a regular tachycardic rhythm. Arterial blood gas analysis confirmed hypoxemia, with a partial pressure of arterial oxygen (PaO_2_) of 61.8 mmHg (reference range: 80-100mmHg). Laboratory investigations revealed anemia, leukocytosis, thrombocytosis, an elevated international normalized ratio (INR), hyponatremia, and elevated inflammatory markers, including C-reactive protein (CRP) and procalcitonin. Admission laboratory values are summarized in Table 1. Electrocardiography showed sinus tachycardia. Chest radiograph revealed a large right-sided hydropneumothorax, with a prominent horizontal air-fluid level and partial collapse of the right lung (Figure 1).

**Table 1:** Summary of laboratory values Reference ranges correspond to the institutional laboratory reference values. CRP: C-reactive protein; INR: international normalized ratio; AST: aspartate aminotransferase; ALT: alanine aminotransferase; -: test not performed

Laboratory parameter	Reference range	Admission	Day 1	Day 3	Day 7	Day 10	Day 14	Day 21
Hemoglobin (g/dL)	11.5-16.5	10.4	10.1	9.6	10.7	11	11.7	12.1
Leukocyte count (cells/μL)	4,000-12,000	14,500	12,700	12,100	11,500	10,000	8,700	9,000
Platelet count (cells/μL)	150,000-400,000	578,000	600,000	514,000	500,000	402,000	280,000	232,000
INR	0.8-1.2	2.6	1.7	1.6	1.5	1.4	1.6	1.2
CRP (mg/L)	<5	199.8	164.7	134.8	112.8	89.3	55.1	5.3
Sodium (mEq/L)	136-145	123	125	127	135	137	142	140
AST (U/L)	10-36	36	30	45	65	40	42	32
ALT (U/L)	10-36	35	38	59	79	58	55	35
Procalcitonin (ng/mL)	<0.05	1.65	-	-	-	-	0.5	-

**Figure 1:**
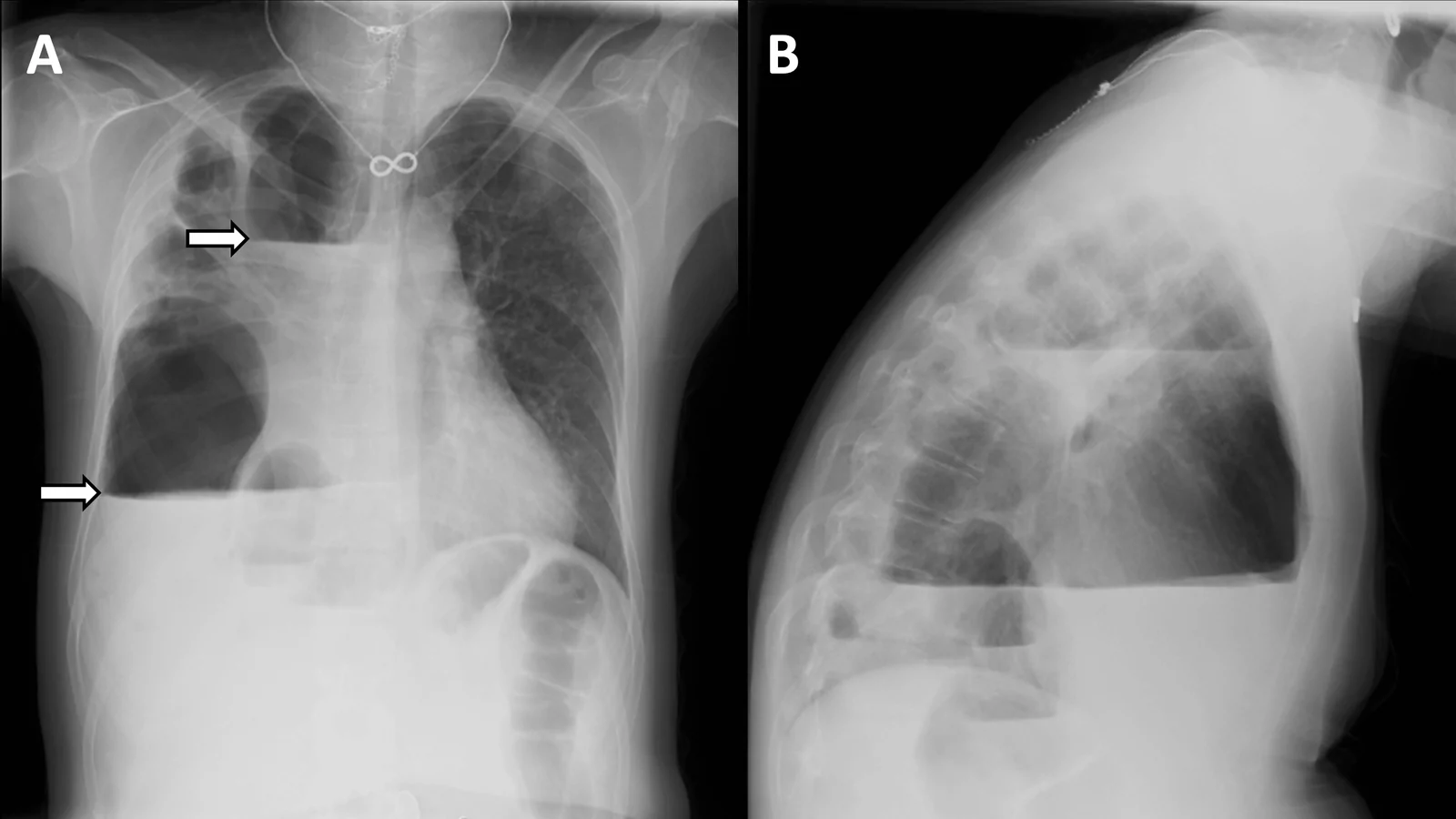
Right-sided hydropneumothorax (admission) Posteroanterior (A) and lateral (B) chest radiographs showing a large right-sided hydropneumothorax with a prominent air-fluid level (white arrows) and associated partial collapse of the right lung.

To further characterize the radiographic findings, a chest computed tomography (CT) was performed, confirming the presence of a right-sided hydropneumothorax and demonstrating scattered areas of consolidation, some with cavitation, and marked architectural distortion (Figure 2).

**Figure 2:**
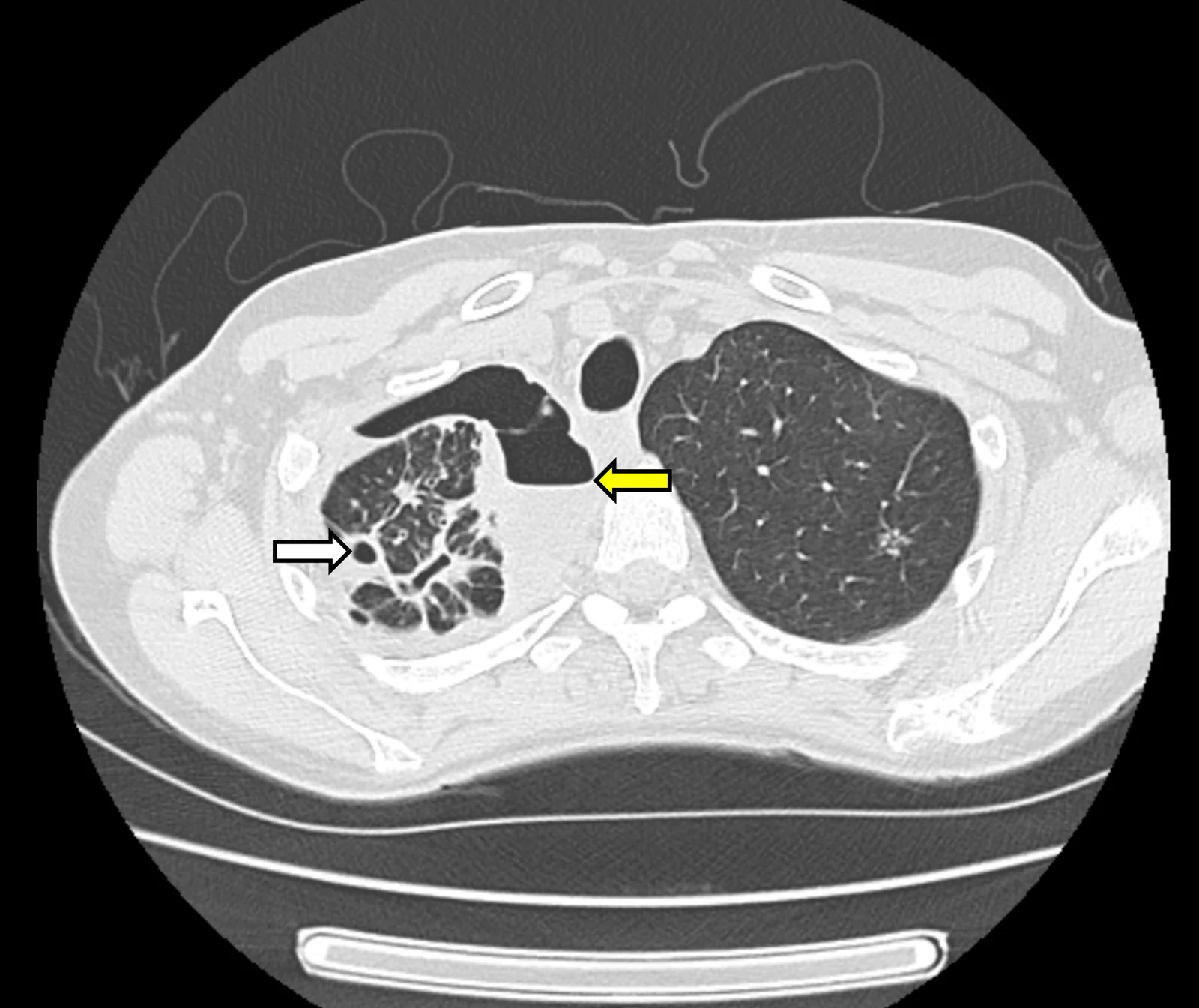
Hydropneumothorax and extensive pulmonary parenchymal abnormalities Axial non-contrast chest CT scan showing a right-sided hydropneumothorax (yellow arrow) and extensive pulmonary parenchymal abnormalities, predominantly involving the right lung and diffusely distributed from the apices to the bases, with scattered areas of consolidation, some with cavitation (white arrow), and marked architectural distortion.

While in the emergency department, supplemental oxygen was initiated via a Venturi mask at an inspired oxygen fraction (FiO_2_) of 40%, and blood cultures and sputum samples were obtained. Despite hypoxemic respiratory failure, the patient remained hemodynamically stable, with no evidence of tension physiology. Given the elevated INR (2.6), chest tube placement was temporarily deferred. In the absence of anticoagulant use or evidence of hepatic dysfunction, the coagulopathy was considered likely related to malnutrition and was corrected with vitamin K. Sputum smear microscopy was positive for acid-fast bacilli, while mycobacterial culture and real-time polymerase chain reaction (PCR) testing using the Anyplex™ MTB/NTMe Real-time Detection assay (Seegene Inc., Seoul, Republic of Korea) were pending. The patient was admitted with a presumptive diagnosis of pulmonary TB with concomitant bacterial superinfection. Empirical antibiotic therapy with amoxicillin/clavulanic acid was initiated, together with standard four-drug antituberculous therapy comprising rifampicin, isoniazid, pyrazinamide, and ethambutol, administered at weight-based doses, and pyridoxine supplementation.

On the following day (day 1), diagnostic thoracentesis was performed, followed by placement of a small-bore pigtail catheter connected to an underwater-seal drainage system. No BPF was directly demonstrated on imaging, and no persistent air leak was observed during pleural drainage. Subsequent imaging demonstrated marked re-expansion of the right lung, with residual apical and basal pleural air-fluid levels and persistent extensive right-sided parenchymal opacities (Figure 3).

**Figure 3:**
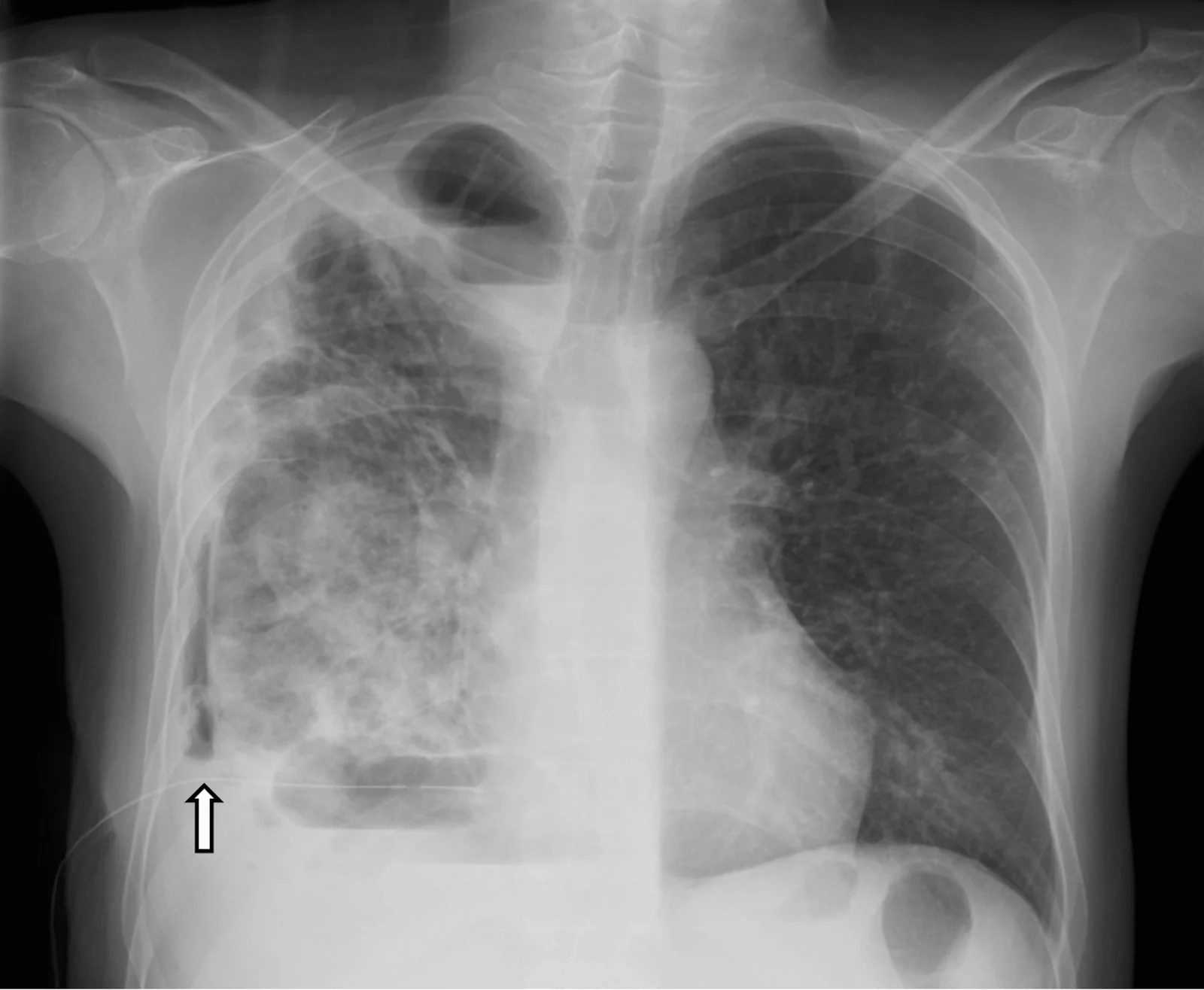
Re-expansion of the right lung (after chest tube placement) Posteroanterior chest radiographs showing marked re-expansion of the right lung with residual apical and basal pleural air-fluid levels and persistent extensive right-sided parenchymal opacities. The chest tube is visible at the right lung base (white arrow).

Pleural fluid analysis revealed a low pH of 7.13, a lymphocyte-predominant differential cell count, a total protein concentration of 4.4 g/dL, a lactate dehydrogenase (LDH) level of 1,876 U/L, and a markedly elevated adenosine deaminase (ADA) level of 119.6 U/L (reference value <30 U/L). Concurrent serum total protein and LDH levels were 5.3 g/dL and 194 U/L, respectively, with the corresponding pleural fluid-to-serum ratios fulfilling Light’s criteria for an exudative effusion.

Anti-TB therapy was well tolerated. At the beginning of hospitalization, molecular testing for anti-TB drug resistance using the Anyplex™ II MTB/MDR Detection assay detected no resistance-associated mutations.

On day 2, sputum molecular testing was positive for the *M. tuberculosis* complex. Molecular testing of the pleural fluid was also positive for the *M. tuberculosis* complex, with later confirmation by mycobacterial culture on Löwenstein-Jensen medium. Blood cultures were negative. Bacterial superinfection remained a presumptive diagnosis, as no bacterial pathogen was microbiologically identified.

The chest tube was removed after 72 hours, following satisfactory re-expansion of the right lung. Two days later, repeat chest CT revealed residual right-sided pneumothorax with persistent architectural distortion (Figure 4). Given the small size of the residual pneumothorax, the absence of respiratory symptoms, and a peripheral oxygen saturation of 97% on room air, conservative management was adopted without repeat pleural drainage or active suction.

**Figure 4:**
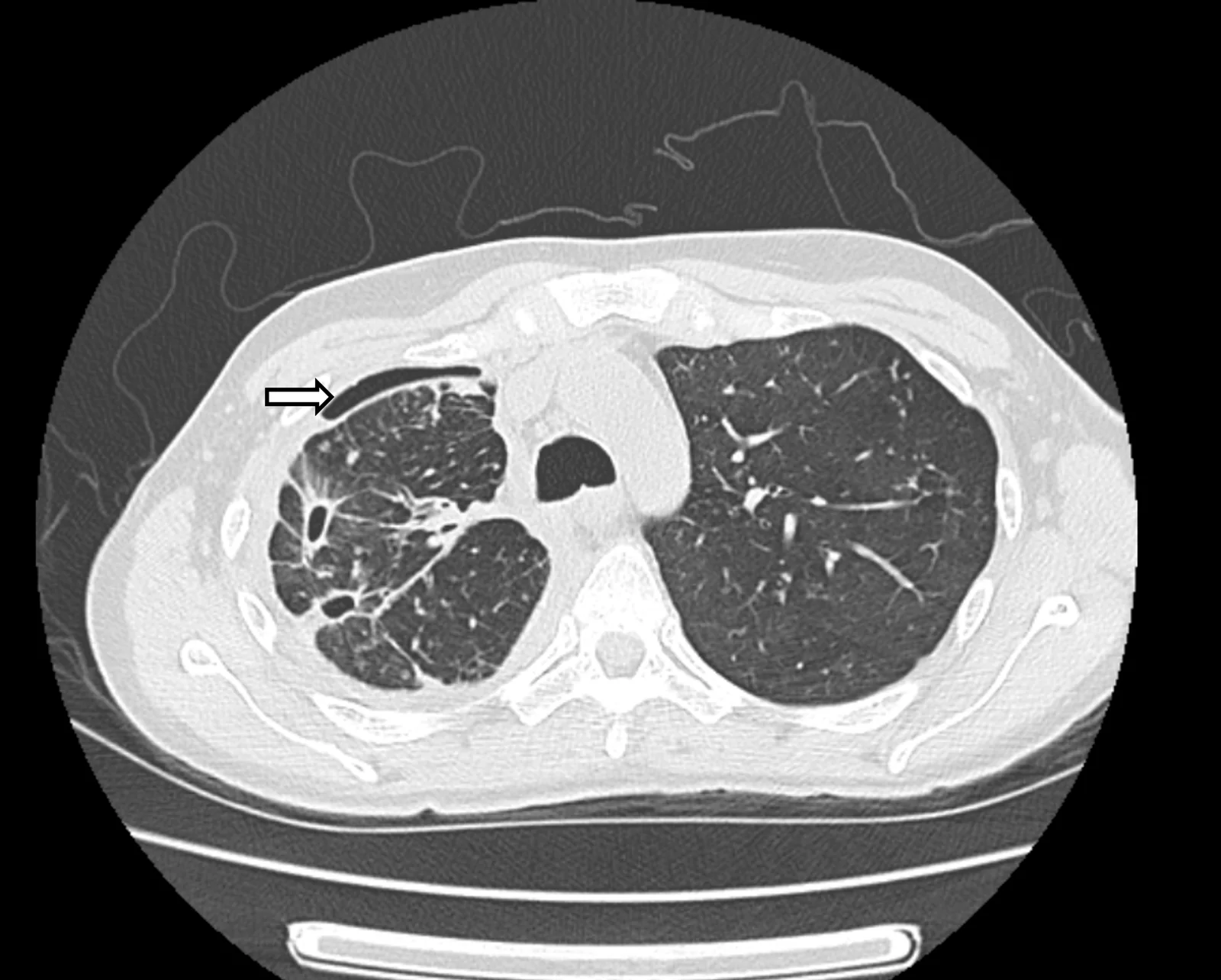
Small residual right-sided pneumothorax (after chest tube removal) Chest CT obtained after chest tube removal showing a small residual right-sided pneumothorax, with persistent architectural distortion and cavitary parenchymal changes in the right lung (white arrow).

The patient showed a favorable clinical course, allowing progressive weaning from supplemental oxygen, which was discontinued on hospital day 5. Inflammatory markers progressively decreased, and sustained apyrexia was achieved. She completed a 14-day course of amoxicillin/clavulanate. The only relevant complication during hospitalization was a mild, transient elevation of liver enzymes with a hepatocellular pattern and predominance of alanine aminotransferase (ALT) over aspartate aminotransferase (AST). The elevation did not meet commonly used biochemical thresholds for clinically significant drug-induced liver injury, and both anti-TB therapy and amoxicillin/clavulanate were continued without interruption or dose adjustment. Liver enzyme levels subsequently normalized spontaneously by the time of discharge. Table 1 summarizes the main laboratory findings over the course of hospitalization.

Follow-up chest CT (Figure 5) obtained before discharge revealed satisfactory re-expansion of the right lung, with a residual right-sided pleural effusion containing small foci of gas and persistent parenchymal abnormalities.

**Figure 5:**
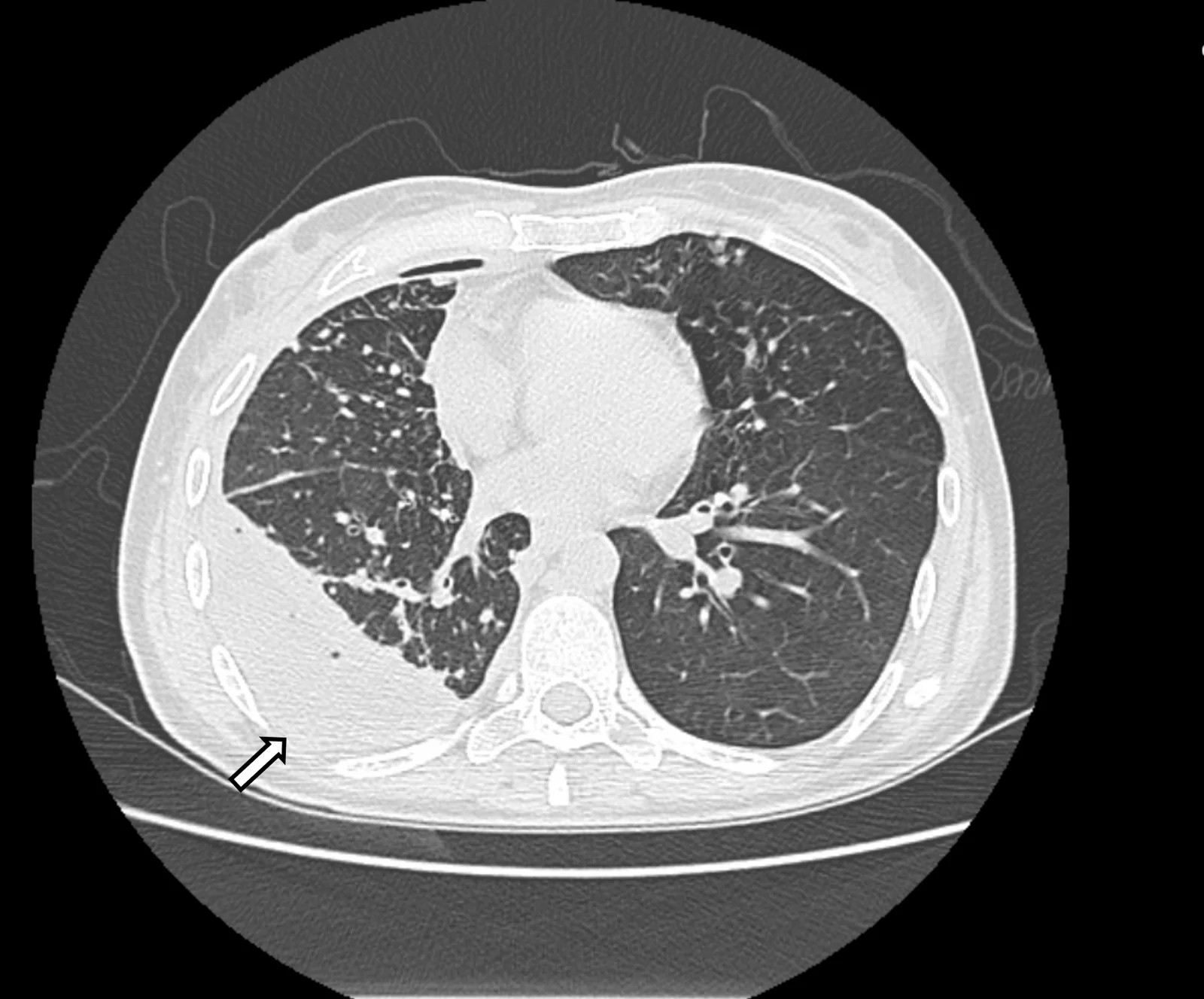
Re-expansion of the right lung with residual right-sided pleural effusion (before discharge) Axial non-contrast chest CT (before discharge) showing re-expansion of the right lung, with a residual right-sided pleural effusion (white arrow) containing small foci of gas and persistent parenchymal abnormalities.

The patient was discharged after 21 days of hospitalization. After discharge, she was referred to the local TB outpatient center for directly observed therapy, as well as to Infectious Diseases and Pulmonology outpatient clinics, the latter for smoking cessation support. Given the diagnosis of pulmonary TB with pleural involvement and no identified drug resistance, she completed a standard six-month anti-TB regimen consisting of a two-month intensive phase with isoniazid, rifampicin, pyrazinamide, and ethambutol, followed by a four-month continuation phase with isoniazid and rifampicin. At the three-month follow-up, the patient showed sustained clinical improvement.

## Discussion

Hydropneumothorax is characterized by the simultaneous presence of air and fluid within the pleural space and is typically recognized on chest radiography by a horizontal air-fluid level. It may arise from several conditions, including infection, trauma, thoracic procedures, malignancy, and the development of a BPF. TB is a well-described infectious cause of hydropneumothorax, although this presentation remains uncommon. Recent evidence characterizes tuberculous hydropneumothorax as a potentially serious complication of pulmonary TB, highlighting the importance of early recognition and appropriate management [3,4].

The pathophysiology of hydropneumothorax in pulmonary TB is closely related to the destructive nature of the disease. Rupture of a subpleural tuberculous cavity or necrotic pulmonary lesion into the pleural space may result in the simultaneous accumulation of air and fluid and may result in a BPF formation [3,4]. Once established, a BPF creates a persistent communication between the tracheobronchial tree and the pleural space and may lead to persistent air leakage, failure of lung re-expansion, pleural infection, and prolonged chest drainage [5,6]. In our patient, chest CT demonstrated extensive bilateral parenchymal involvement, predominantly affecting the right lung, with scattered areas of consolidation, some with cavitation, marked architectural distortion, and a right-sided hydropneumothorax. These findings provide a plausible anatomical substrate for communication between a peripheral tuberculous lesion and the pleural space, although a BPF was not directly demonstrated.

The differential diagnosis of hydropneumothorax depends on the clinical setting and includes bacterial empyema or pyopneumothorax, necrotizing pneumonia or lung abscess with pleural rupture, malignancy, trauma, and iatrogenic causes. In the present case, the absence of previous thoracic surgery or trauma, together with fever, productive cough, constitutional symptoms, extensive bilateral parenchymal abnormalities, cavitary lesions, and a sputum smear positive for acid-fast bacilli, strongly supported an infectious etiology consistent with pulmonary TB. Moreover, molecular testing of both sputum and pleural fluid was positive for *M. tuberculosis* complex, confirming pulmonary TB with pleural involvement. This case therefore emphasizes the importance of considering TB in the etiological investigation of spontaneous hydropneumothorax when compatible clinical and radiological features are present, even though other infectious and non-infectious causes must be excluded.

Management of tuberculous hydropneumothorax requires treatment of both the underlying mycobacterial infection and the associated pleural complication. Antituberculous therapy should be initiated according to microbiological results and drug susceptibility, while pleural drainage is generally required in patients with a significant pneumothorax, respiratory compromise, infected pleural fluid, or impaired lung expansion. Persistent air leakage, failure of lung re-expansion, pleural thickening, or an established BPF may require prolonged drainage and, in selected patients, bronchoscopic or surgical intervention [3-6]. In a recent prospective observational study of 50 patients with microbiologically confirmed pulmonary TB presenting with pneumothorax or hydropneumothorax, Kumar et al. reported a mean lung expansion time of 2.92 ± 2.16 weeks; complete radiological expansion was achieved in 76% of patients within eight weeks, while 24% required referral for cardiothoracic surgical evaluation [3].

Our case is noteworthy for the rapid and satisfactory re-expansion of the right lung following chest tube drainage, despite the extensive parenchymal and cavitary involvement and the large hydropneumothorax at presentation. In contrast to several recently reported cases of tuberculous hydropneumothorax complicated by BPF, persistent air leakage, incomplete lung re-expansion, or the need for prolonged drainage or surgical intervention [7-9], our patient had only a small residual pneumothorax after chest tube removal, with no subsequent recurrence requiring repeat pleural drainage or further pleural complications. This favorable clinical course illustrates the heterogeneous evolution of tuberculous hydropneumothorax and highlights that even extensive radiological disease may respond favorably to appropriate pleural drainage combined with anti-TB therapy. TB should therefore remain an important differential diagnosis in patients presenting with spontaneous hydropneumothorax and compatible constitutional, respiratory, and radiological findings. Early recognition and appropriate management of both the pleural complication and the underlying infection may facilitate lung re-expansion and reduce the need for more invasive interventions.

## Conclusions

Although uncommon, hydropneumothorax is a clinically relevant pleural complication of pulmonary TB and may arise from communication between a peripheral tuberculous lesion and the pleural space, potentially through a BPF. In the present case, however, no BPF was directly demonstrated, and this mechanism therefore remains hypothetical. Management should address both the underlying infection and the pleural complication, with pleural drainage considered in the presence of significant pneumothorax, respiratory compromise, infected pleural fluid, or impaired lung expansion. In this case, anti-TB therapy combined with pleural drainage was associated with satisfactory lung re-expansion without the need for further pleural intervention or surgery. Recognition of this presentation may facilitate timely diagnosis and appropriate management, ultimately contributing to favorable clinical outcomes.
